# Efficacy evaluation of Veeralin LN, a PBO-incorporated alpha-cypermethrin long-lasting insecticidal net against *Anopheles culicifacies* in experimental huts in Odisha State

**DOI:** 10.1186/s12936-020-03480-6

**Published:** 2020-11-10

**Authors:** Gunasekaran Kasinathan, Sudhansu Sekhar Sahu, Nallan Krishnamoorthy, Mohammed Mustafa Baig, Sonia Thankachy, Smrutidhara Dash, Swaminathan Subramanian, Purushothaman Jambulingam

**Affiliations:** Indian Council of Medical Research-Vector Control Research Centre, Medical Complex, Indira Nagar, Puducherry, 605006 India

**Keywords:** Alpha**-**cypermethrin, *Anopheles culicifacies*, Experimental huts, Piperonyl butoxide, Veeralin

## Abstract

**Background:**

The success of malaria control using long-lasting insecticidal nets (LLINs) is threatened by pyrethroid resistance developed by the malaria vectors, worldwide. To combat the resistance, synergist piperonyl butoxide (PBO) incorporated LLINs is one of the available options. In the current phase II hut trial, the efficacy of Veeralin^®^LN (an alpha-cypermethrin and PBO-incorporated net) was evaluated against *Anopheles culicifacies*, a pyrethroid resistant malaria vector.

**Methods:**

The performance of Veeralin^®^LN was compared with MAGNet^®^LN and untreated net in reducing the entry, induced exit, mortality and blood feeding inhibition of target vector species.

**Results:**

The performance of Veeralin was equal to MAGNet in terms of reducing hut entry, inhibiting blood feeding and inducing exophily, and with regard to causing mortality Veeralin was better than MAGNet. When compared to untreated net, a significant reduction in hut entry and blood feeding and an increase in exophily and mortality were observed with Veeralin. In cone bioassays, unwashed Veeralin caused > 80% mortality of *An. culicifacies*.

**Conclusions:**

Veeralin performed equal to (entry, exit, feeding) or better than (mortality in huts and cone bioassays) MAGNet and could be an effective tool against pyrethroid resistant malaria vectors.

## Background

Controlling the malaria vectors with indoor residual spraying (IRS) of insecticides and/or long-lasting insecticidal nets (LLINs) is the major approach used for preventing malaria transmission [1]. Recent studies established that up-scaling of these tools has paved way for malaria reduction in many countries [1]. Pyrethroids are the insecticides primarily used in various formulations for mosquito control especially in LLINs [1]. Due to the extended use of LLINs and IRS, increased spread of vector resistance to different insecticides has been reported worldwide, which could compromise the effectiveness of these interventions [1–5], thereby limiting the use of LLINs for malaria control in many parts of the world [1]. Although, new molecules with different modes of action have been pre-qualified by the World Health Organization (WHO) for LLIN treatment, synthetic pyrethroids are the primary class of insecticide licensed for treating bed nets and other netting materials used for personal protection [6]. Therefore, there is a need for preserving the effectiveness of pyrethroid-based LLINs/vector control tools [1]. The efficacy of LLINs could be sustained against malaria vectors for a minimum of three years without any re-treatment with a good LLIN technology [7–9].

Current interest of the manufacturers is to focus on developing innovative control strategies that can combat pyrethroid resistance [10, 11]. For controlling pyrethroid resistant mosquitoes, the only available alternative is piperonyl butoxide (PBO) incorporated LLINs which are having increased efficacy against pyrethroid-resistant malaria vectors [9, 12]. Further, this is an insecticide resistance management approach and a potential means to fight against insecticide resistance [1]. The PBO-LLINs were shown to be effective against some resistant populations of *Anopheles* in experimental hut trials [11, 13]. As there is a need to look for new products that are efficient against pyrethroid-resistance malaria vectors, PBO-incorporated pyrethroid nets have been approved with an interim recommendation [9, 14] pre-qualified by the WHO as a new class of vector control products [15]. Two PBO-LLINs, DawaPlus 3.0 and DawaPlus 4.0, have been granted an interim recommendation in 2017 [14], and five more PBO-LLINs, namely PermaNet 3.0, Olyset Plus, Veeralin, Tsara Boost and Tsara Plus, have been pre-qualified by the WHO in 2018 [6]. The current article summarizes the phase II evaluation of Veeralin^®^LN conducted in a setting (an area) where the malaria vector, *An. culicifacies* was resistant to synthetic pyrethroids [16]. The performance of the Veeralin LLIN was compared with the MAGNet LLIN, a WHO-pre-qualified alpha-cypermethrin LLIN [6] (positive control) and with a negative control, an untreated net. The efficacy of Veeralin was evaluated in experimental huts that simulate domestic habitations, against a free flying wild population of *An. culicifacies* in terms of preventing hut entry, inducing exit, inhibiting blood-feeding and causing mortality in a tribal district of Odisha State, India.

## Methods

### Trial site and study design

The current Phase II trial was carried out in experimental huts located at the village, Kandhaguda of Malkangiri District (17° 45′ N to 18° 40′ N and 81° 10′ E to 82° 00′ E) in Odisha State. The major human malaria parasite species prevailing in this area was *Plasmodium falciparum.* Transmission of malaria peaks in the district during rainy (July–August) and cooler months (November–December). The two vector species that transmit malaria in this area were *Anopheles fluviatilis* and *An. culicifacies*. *Anopheles fluviatilis* was highly susceptible to synthetic pyrethroids whereas, *An. culicifacies* was found to be resistant [16] with moderate resistance intensity/level [17]. The specifications of the hut construction have been described elsewhere [18].

### Description of the test product

In the test LLIN, Veeralin^®^LN, manufactured by VKA Polymers, Tamil Nadu, India, the pyrethroid insecticide, alpha-cypermethrin was integrated into monofilament polyethylene fibres of 130 denier at a target dose of 6.0 g AI/kg, equivalent to 216 mg AI/m^2^ of insecticide, and 2.2 g/kg of PBO, equivalent to 79.2 mg/m^2^. In the current trial, six arms viz., Veeralin unwashed and washed 20 times, MAGNet unwashed and 20 times washed (positive control), untreated net (negative control) and 25 times washed Veeralin^®^LN were compared. All the trial nets (size 180 × 160 × 150 cms) were sponsored by VKA Polymers. Each arm had six replicate nets and every night different replicate nets of the same arm were tested in each hut for each week. Chemical analysis and cone bioassays before any wash and after 20 or 25 washes were carried out on additional nets.

### Washing and cone-bioassays

Nets were washed according to the WHO washing protocol [8]. Cone bioassays were performed on the net surfaces of the additional nets included for this purpose in each treatment arm. Before any wash and after 20 or 25 washes, for the cone bioassays, laboratory-reared, pyrethroid susceptible *Anopheles stephensi* mosquitoes were used as *An. culicifacies*, the target test mosquito species was not available at the rearing facility of Indian Council of Medical Research (ICMR)—Vector Control Research Centre (VCRC), Puducherry, where washing of nets was carried out. Bioassays were also carried out at the field site i.e., after the evaluation in the experimental huts, exposing wild caught pyrethroid resistant *An. culicifacies* and pyrethroid susceptible *Anopheles jeyporiensis* on one net selected randomly from the six replicate nets of each arm used in the experimental huts. On each net, bioassays were performed on all the five panels (one roof and four side panels). Two cone tests were carried out on each panel, releasing five mosquitoes per cone test. Thus, a total of 50 mosquitoes each of the susceptible and resistant species were tested per net. The mosquitoes were exposed for 3 min and after the exposure, they were held for 24 h at temperature 27 ± 2 °C and humidity 75 ± 10%. During the holding time, the mosquitoes were provided with sugar solution for feeding. The mortality was recorded after 24 h.

### Analysis for insecticide content in nets

To determine the alpha-cypermethrin and PBO content in the treatment arms, chemical analysis of LLIN samples was done, prior to and after washing 20 or 25 times and after hut evaluation. Prior to wash, 5 pieces, one (30 × 30 cm) from each of the 5 positions, were cut and removed from one of the three additional nets of each arm. For chemical analysis, net samples were also obtained from 20 times washed (2nd additional net) and 25 times washed net (3rd additional net), and similarly from unwashed arms. After experimental hut study, from each arm, one of the six replicate nets used in the huts was randomly chosen for the analysis. The samples, after packing in aluminium foil, were labelled and stored at + 4 °C and sent to the WHO Collaborating Centre, Gembloux, Belgium, for analysing the active ingredient content of alpha-cypermethrin and PBO.

### Selection of volunteers for hut evaluation

For sleeping in experimental huts, 12 adult volunteers, two (preferably husband and wife) for each hut, were selected from the village through organizing a meeting.To engage human participants in the hut evaluation, clearance was obtained from the ICMR-VCRC Human Ethics Committee. Prior written consent was obtained from the volunteers for their participation. The volunteers were told to sleep under the net using the bed materials that were allotted to them from 19.00 to 05.30 h with a small interval for dinner and not to make any fire or smoke inside the hut.

### Net preparation and rotation of nets and volunteers in experimental huts

Before starting the evaluation, in each of the six replicate nets of all the arms, a total of six holes (each one: 4 cm × 4 cm) were made to give an effect of a torn net. Six teams, each with two sleepers, were formed. The nets and the sleepers were rotated as per the Latin square design to reduce the differences due to attractiveness of huts and volunteers. One treatment arm was tested for one week in each hut with one replicate net each day. The sleepers were rotated among the huts daily, whereas, the arms were rotated weekly. The collection and processing of mosquitoes have been described elsewhere [18].

### Data collection and analysis

The number of mosquitoes entered, exited, dead inside the hut and the number that succeeded to feed on volunteers were calculated for each arm by compiling the data collected over 12 weeks. The day-wise entry of *An. culicifacies* in each arm was recorded. As the variance was higher than the mean; negative binomial regression analysis (using Stata software, Version 14) was done by taking the number collected as dependent variable and the experimental arms as independent variable. The number of *An. culicifacies* exited, blood fed and dead after 24 h (total mortality) day-wise and arm-wise were analysed using logistic regression (using SPSS 16.0) by taking mosquitoes exited, fed and dead (total mortality) as dependent variables and arms as categorical covariates. For overall comparison, the negative control (untreated net) was kept as reference category. Further comparisons between the treated arms were made by removing negative control from the grouped data and holding the positive control (unwashed MAGNet) as reference category. The mean alpha-cypermethrin and PBO content in the net samples prior to any wash, after washing and after hut evaluation were compared using one-way ANOVA followed by a rank (LSD) test.

## Results

### Species composition in the experimental huts

In total, 72 collections, completing two rotations (six weeks to complete one rotation) across all treatments, were done for each of the six arms in the experimental huts. A total of 650 mosquitoes were collected. Among them, *An*. *culicifacies* formed 69.4%, *An. fluviatilis* 2.3%, and *Anopheles subpictus* and *Anopheles vagus* (non-vectors) together formed 19.5%, and 8.8% was culicines. As the trial was carried out at the end of the rainy season, the number of *An. fluviatilis* collected was very small. Therefore, further analysis was restricted to *An. culicifacies*, which was resistant to alpha-cypermethrin.

### Entry of *Anopheles culicifacies* into the huts

The mean number ± SE of *An*. *culicifacies* that entered the huts (entry) with unwashed Veeralin, 20 times washed Veeralin, unwashed MAGNet, 20 times washed MAGNet, untreated nets and Veeralin washed 25 times was 0.47 ± 0.11, 0.56 ± 0.08, 0.51 ± 0.10, 0.46 ± 0.08, 3.61 ± 0.28, and 0.65 ± 0.11, respectively from 72 collections during 12 weeks (Table 1). Out of 72 attempts of mosquito collections, on six occasions, there was no entry of *An*. *culicifacies* into the huts with untreated net, whereas, in the five treated arms; MAGNet without any wash, MAGNet after 20 washes, Veeralin without any wash, Veeralin after 20 and 25 washes, zero entry was recorded on 47, 47, 52, 40 and 43 occasions, respectively. The hut entry was significantly lower in the five treated arms (p < 0.05) than the untreated one. It was noted from the incidence rate ratio (IRR) that, among the treated arms, the hut entry was the minimum with MAGNet washed 20 times followed by unwashed Veeralin. However, no significant difference was seen in the entry between the five treated arms indicating a similar deterrent effect of all the treated arms (Table 1).

**Table 1:** Insecticidal efficacy of different arms on entry, exit, mortality and blood-feeding of *An. culicifacies* collected in the experimental hut trials

	Unwashed Veeralin LN	Veeralin LN washed 20 times	Unwashed MAGNet LN	MAGNet LN washed 20 times	Untreated polyester net	Veeralin LN washed 25 times
Number of collections	72	72	72	72	72	72
Total females entered	34	40	37	33	260	47
IRR (95% CI)	0.130 (0.088–0.193)	0.153 (0.106–0.222)	0.142 (0.097–0.207)	0.126 (0.085–0.188)	1.00^a^	0.180 (0.127–0.256)
p	0.000	0.000	0.000	0.000	–	0.000
Total females exit in veranda trap (Exit rate in %)	18 (52.9)	23 (57.5)	15 (40.5)	17 (51.5)	65 (25.0)	24 (51.1)
Odds ratio (95% CI)	3.375 (1.627–7.001)	4.059 (2.042–8.067)	2.045 (1.002–4.176)	3.187 (1.524–6.668)	1.00^a^	3.130 (1.655–5.920)
p	0.001	0.000	0.049	0.002	–	0.000
Total females blood fed (% blood feeding)	23 (67.6)	22 (55.0)	21 (56.8)	19 (57.6)	246 (94.6)	29 (61.7)
% Blood feeding inhibition	28.5	41.9	40.0	39.1	–	34.8
Odds ratio (95% CI)	0.128 (0.053–0.311)	0.075 (0.033–0.169)	0.080 (0.035–0.185)	0.083 (0.035–0.197)	1.00^a^	0.083 (0.038–0.180)
p	0.000	0.000	0.000	0.000	–	0.000
% Personal protection	90.7	91.1	91.5	92.3	0.0	88.2
Total females dead (% total mortality)	32 (94.1)	32 (80.0)	25 (67.6)	22 (66.7)	0 (0.0)	26 (55.3)
Odds ratio (95% CI)	0.130 (0.027–0.636)	0.521 (0.185–1.468)	1.00^a^	1.042 (0.384–2.828)		1.683 (0.686–4.126)
p	0.012	0.217	–	0.936		0.255
% Mass killing effect	12.3	12.3	9.6	8.5	0.0	10.0

^a^Reference category

### Exit from the huts

The five treated arms induced significantly a higher exophily (p < 0.05) than the untreated arm. The exit rate of *An. culicifacies* was higher with Veeralin washed 20 times (57.5%) followed by unwashed Veeralin (52.9%). However, the 95% CI for the odds ratios showed no significant difference in the exit rate between the treated arms (Table 1). By taking the positive control (unwashed MAGNet) as reference category (by excluding the negative control), no significant difference was observed in exit rates of *An. culicifacies* among the five treated arms (logistic regression: likelihood ratio based χ^2^ = 2.358, df = 4, p = 0.67).

### Inhibition of blood feeding and personal protection

The lowest blood feeding (i.e. the highest feeding inhibition) was observed with Veeralin washed 20 times (55.0%) followed by unwashed MAGNet (56.8%) (Table1). Overall, the feeding rate among the six experiment arms greatly differed (χ^2^ = 89.435, df = 5, p < 0.05). Compared to untreated arm, all treated arms recorded a significantly reduced feeding rate. Between the treatment arms, no significant difference was found, as shown by 95% CI for the Odds ratios. Personal protection determines the level of protection offered by each treatment arm. Among the five treatment arms, the personal protective effect was the maximum with MAGNet washed 20 times (92.3%) followed by unwashed MAGNet (91.5%), Veeralin washed 20 times (91.1%), unwashed Veeralin (90.7%) and Veeralin washed 25 times (88.2%).

### Mortality and mass killing effect

Higher mortality was seen in all treatment arms (no mortality with untreated net), the maximum being with unwashed Veeralin (94.1%) (Table1). Overall, the mortality differed significantly among the five treatment arms (χ^2^ = 18.979, df = 4, p = 0.001) (Table 1). Veeralin washed 20 and 25 times and MAGNet washed 20 times did not differ significantly from unwashed MAGNet, the positive control, in terms of causing mortality (p > 0.05) except unwashed Veeralin that caused a significantly greater mortality (p = 0.012). Overall, the mass killing effect varied from 8.5% (MAGNet washed 20 times) to 12.3 (both unwashed and 20 times washed Veeralin) (Table 1).

### Cone bioassay tests

Before any wash, after 20 or 25 washes, while the treatment arms produced 100% mortality of the pyrethroid susceptible *An. stephensi*, the untreated nets caused zero mortality. After hut evaluation, only unwashed Veeralin caused > 80% mortality of *An. culicifacies*, and the mortality with the other treated arms was < 80% (Table 2). The bio-assays conducted with pyrethroid susceptible *An. jeyporiensis* on the treated arms after hut evaluation showed 100% mortality.

**Table 2:** Percentage of mortality in WHO cone-bioassays

Experiment arms	Bioassays before hut evaluation with *An. stephensi*									Bioassays after hut evaluation					
	Before any wash			After 20 washes			After 25 washes			With *An. culicifacies*			With *An. jeyporiensis*		
	NE	CM (%)	95% CI	NE	CM (%)	95% CI	NE	CM (%)	95% CI	NE	CM (%)	95% CI	NE	CM (%)	95% CI
Unwashed Veeralin LN	50	100	92.9–100.0	–	–	–	–	–	–	50	86	73.3–94.2	50	100	92.9–100.0
Veeralin LN washed 20 times	50	100	92.9–100.0	50	100	92.9–100.0	–	–	–	50	76	61.8–86.9	50	100	92.9–100.0
Unwashed MAGNet	50	100	92.9–100.0	–	–	–	–	–	–	50	72	57.5–83.8	50	100	92.9–100.0
MAGNet washed 20 times	50	100	92.9–100.0	50	100	92.9–100.0	–	–	–	50	78	64.0–88.5	50	100	92.9–100.0
Untreated polyester	50	0	0.0–7.1	–	–	–	–	–	–	50	0	0.0–7.1	50	0	0.0–7.1
(Negative control)									–						
Veeralin LN washed 25 times	50	100	92.9–100.0	–	–	–	50	100	92.9–100.0	50	68	53.3–80.5	50	100	92.9–100.0

*NE *number exposed, *CM *corrected mortality, *95% CI* 95% confidence intervals based on exact binomial probability distribution

### Chemical analysis

The average base-line (wash 0) content of alpha-cypermethrin in the net samples of the three Veeralin arms viz., unwashed Veeralin, Veeralin washed 20 times and 25 times was 5.61 ± 0.12 (SD), 5.61 ± 0.08 and 5.63 ± 0.06 g/kg, and the nets complied with the target dose of 6.0 g/kg ± 25% (4.5 g/kg-7.5 g/kg). The alpha-cypermethrin content after 20 and 25 washes was 5.44 ± 0.07 g/kg and 5.49 ± 0.11 g/kg, corresponding to an overall alpha-cypermethrin retention of 97% and 98%, respectively. After hut evaluation, the alpha-cypermethrin content was 5.59 ± 0.06, 5.52 ± 0.05 and 5.44 ± 0.12 g/kg in Veeralin unwashed and washed 20 times and 25 times, respectively. After washing (20 or 25 times), there was a decline in alpha-cypermethrin content (p < 0.05 by One-way ANOVA) compared to the base-line concentration, but, within the specifications of ± 25%. No further diminishing of active ingredient content was observed after the hut trial (p > 0.05).

In the two MAGNet arms, unwashed and 20 times washed, the initial alpha-cypermethrin content was 5.82 ± 0.05 and 5.80 ± 0.05 g/kg and that met comply with the target dose of 5.8 g/kg ± 25% (4.4–7.3 g/kg). Compared to the initial level, the active ingredient concentration reduced to 5.46 ± g/kg after 20 washes (p < 0.05), but within the accepted margin of ± 25%. The corresponding active ingredient retention was 94%. After the hut evaluation, alpha-cypermethrin content did not decrease significantly (p > 0.05), as it was 5.59 ± 0.08 for MAGNet unwashed and 5.36 ± 0.13 g/kg for MAGNet washed 20 times.

The mean base-line PBO content in the three Veeralin arms (2.47 ± 0.02, 2.45 ± 0.03 and 2.42 ± 0.05 g/kg, respectively) were within the acceptable range of the target dose of 2.2 g/kg ± 25% (1.7–2.8 g/kg). After 20 and 25 washes, there was a decrease in PBO content to 2.01 ± 0.02 g/kg and 2.01 ± 0.06 g/kg (p < 0.05 by one-way ANOVA); however, the content was within the specification of ± 25%. The overall retention of PBO content after washing was 82% and 83%, respectively. After hut trial, while no further decline in PBO content (2.02 ± 0.03 g/kg) was noticed in Veeralin washed 20 times (p > 0.05), there was a decrease in 25 times washed Veeralin (1.94 ± 0.04 g/kg)(p < 0.05). In unwashed Veeralin, after hut trial, PBO content declined to 2.31 ± 0.03 g/kg compared to the base-line (p < 0.05). However, in all cases, the PBO content was within the acceptable range of ± 25% (1.7–2.8 g/kg).

## Discussion

The current study assessed the response of *An. culicifacies* to alpha-cypermethrin + PBO nets in comparison to only alpha-cypermethrin nets in reducing the entry and blood-feeding and in inducing exophily and mortality in experimental huts. There was no entry of *An*. *culicifacies* into the huts on many occasions when the volunteers slept under the treated nets (of the five treatment arms), signifying a higher deterrent effect of the pyrethroid insecticide, alpha-cypermethrin used in the treated nets. In spite of the holes made, the deterrent effect was also realized from the 10 alive *An. culicifacies* collected inside the untreated nets whereas, only one alive and one dead were found under the nets of the five treated arms. Thus, all the treated arms significantly reduced the entry of *An. culicifacies* in to the huts compared to the untreated arm. Further, the washed treatment arms did not differ significantly from the unwashed with regard to hut entry indicating no impact of washing of Veeralin LN/MAGNet LN on their deterrent effect*.*

Considerable exit of *An. culicifacies* (25%) was also noticed from the huts with untreated net (negative control), which might be due to its innate behaviour. An experimental hut study conducted recently in the same area showed that the exit rate (induced exophily) of *An. culicifacies* in the untreated arm was 23.9% [19]. However, a higher exophily (40.5–57.5%) was recorded with the treated arms, but between them, there was no significant difference. Veeralin without any wash and after 25 washes induced a comparable exophily, indicating the retention of insecticide even after 25 washes.

Among the treated arms, Veeralin washed 20 times (41.9%) effected the maximum inhibition of blood feeding followed by MAGNet unwashed (40.0%). The feeding rate was significantly lower in all the treatment arms than the untreated arm. When compared to the positive control, the blood feeding inhibition in the treatment arms did not differ significantly.

The total mortality of *An. culicifacies* in the five treated arms varied from 55.3 to 94.1%, the maximum was by unwashed Veeralin and that was much higher than the mortality caused by the positive control. Further, the cone-bioassay results showed that only unwashed Veeralin caused > 80% mortality of *An. culicifacies* (pyrethroid resistant) at the end of hut evaluation. However, the enhanced mortality produced by alpha-cypermethrin + PBO LLINs was not sustained after 20 washes. Similar results were obtained in experimental hut trials in Benin, Burkina Faso and Cameroon, where deltamethrin-PBO LLINs produced significantly a higher mortality of *An. gambiae sensu lato* (*s.l.*) (pyrethroid resistant) compared to non-PBOLLINs, with higher mortality observed with unwashed LLINs [10, 13].

The cone-bioassays conducted during the current trial with pyrethroid resistant *An. culicifacies* after hut evaluation showed that unwashed Veeralin met the efficacy criteria of > 80% mortality [8]. After washing, both Veeralin and positive control net (MAGNet) showed < 80% mortality. It was reported that the PBO content in one product with WHOPES interim recommendation remained stable after 10 laboratory washes, indicating that no PBO was being released after 10 washes and, therefore, was not bio-available on the net surface [20]. When pyrethroid susceptible mosquitoes (*An. stephensi* and *An. jeyporiensis*) were exposed to all the five treatment arms, 100% mortality was observed. It is known that against susceptible mosquito populations, pyrethroid-PBO nets may not have any effect [21], but combination or mixture LLINs with PBO could be effective against resistant mosquitoes whose resistance is based on oxidative metabolism [11]. Overall, the performance of unwashed Veeralin was significantly higher than the negative control (untreated net) and better than the positive control (MAGNet) in causing mortality of *An. culicifacies* in experimental huts. The chemical analysis though, indicated a loss of insecticide and PBO after 20 or 25 washes, their content, even after the loss, remained within the accepted range of ± 25%. Therefore, the marked increase in mortality noticed with Veeralin LN was probably due to the addition of the synergist, PBO, which is known to inhibit the activity of the enzymes that are responsible detoxification of pyrethroids [22]. Nevertheless, it was not possible to attain a complete control of the pyrethroid resistant *An. culicifacies* with Veeralin in experimental huts and this could be due to the presence of different resistance mechanisms that are not affected by the synergist, PBO [23].

Phase II LLIN evaluations mainly focus on two entomological parameters, blood feeding inhibition and mortality, which are crucial for obtaining interim recommendation/pre-qualification [24]. In the current study, Veeralin after 20 or 25 washes produced similar effect on the entomological parameters compared to the other treated arms, except mortality where unwashed Veeralin caused a higher mortality than washed ones. This clearly showed that Veeralin was comparable with/better than MAGNet.

Experimental hut studies conducted in West and Central Africa, Tanzania and Togo with high pyrethroid resistance showed that pyrethroid-PBO nets performed better than standard-LLINs [9, 10, 25], whereas in moderate to low pyrethroid resistant areas, there was little or no difference in the effect of unwashed/washed pyrethroid-PBO nets compared to unwashed/washed standard-LLINs [19, 25]. This may be due to the wide variation in insecticide resistance levels in malaria vector species. However, in the current experimental hut trial, the performance of Veeralin LN was comparable to MAGNet LN (positive control) in causing deterrence, inducing exophily and inhibiting blood feeding and better in terms of causing mortality of the pyrethroid resistant *An*. *culicifacies*.

## Conclusions

In the current study, Veeralin LN performed equal to (entry, exit, feeding) or better than (mortality in huts) the reference LN (MAGNet) and fulfilled the WHO-PQ criteria of Phase II evaluation of LLINs. In view of increasing development of pyrethroid resistance in malaria vectors, PBO incorporated Veeralin LN could be an option to be considered for malaria control after fully qualified as a vector control product by the WHO.

## Data Availability

The data sets generated and/or analysed during the current study are available from the corresponding author on reasonable request.

## Abbreviations

LLIN: Long-lasting insecticidal net
PBO: Piperonyl butoxide
IRS: Indoor residual spraying
WHO: World Health Organization
WHOPES: World Health Organization Pesticide Evaluation Scheme
ICMR-VCRC: Indian Council of Medical Research-Vector Control Research Centre
SE: Standard error
IRR: Incidence rate ratio
CI: Confidence intervals
df: Degrees of freedom
